# The major worldwide stress of healthcare professionals during the first wave of the COVID-19 pandemic – the international COVISTRESS survey

**DOI:** 10.1371/journal.pone.0257840

**Published:** 2021-10-06

**Authors:** Sébastien Couarraze, Louis Delamarre, Fouad Marhar, Binh Quach, Jiao Jiao, Raimundo Avilés Dorlhiac, Foued Saadaoui, Andy Su-I Liu, Benoït Dubuis, Samuel Antunes, Nicolas Andant, Bruno Pereira, Ukadike C. Ugbolue, Julien S. Baker, The COVISTRESS network, Maëlys Clinchamps, Frédéric Dutheil

**Affiliations:** 1 Department of Anesthesiology and Critical Care, University Hospital of Toulouse, University Toulouse 3-Paul Sabatier, Toulouse, France; 2 UMR EFTS, Université Toulouse 2 –Jean Jaurès, Toulouse, France; 3 Université Clermont Auvergne, CNRS, LaPSCo, Physiological and Psychosocial Stress, University Hospital of Toulouse, CHU Toulouse, France; 4 Hong Kong Baptist University, Physical Education and Health, Centre for Health and Exercise Science Research, Kowloon Tong, Hong Kong; 5 Universidad Finis-Terrae, Hospital Dr. Luis-Valentìn-Ferrada, El-Carmen, Maipù, Chile; 6 King Abdulaziz University, Saudi Arabia, College of Sciences and Theoretical Studies, Riyadh, Saudi Arabia; 7 University of Taipei, Exercise and Health Science, Taipei, Taiwan; 8 Université de Genève, UNIGE, Fondation INARTIS, Genève, Switzerland; 9 ISPA—Instituto Universitário, Ordem dos Psicólogos Portugueses, APPsyCI—Applied Psychology Research Center Capabilities & Inclusion, Lisboa, Portugal; 10 University Hospital of Clermont Ferrand, CHU Clermont-Ferrand, Clinical Research and Innovation Direction—Biostatistics, Clermont-Ferrand, France; 11 University of the West of Scotland, Institute for Clinical Exercise & Health Science, School of Health and Life Sciences, South Lanarkshire, Scotland, UK; 12 University Hospital of Clermont-Ferrand, CHU Clermont-Ferrand, Preventive and Occupational Medicine, Clermont-Ferrand, France; 13 Université Clermont Auvergne, CNRS, LaPSCo, Physiological and Psychosocial Stress, University Hospital of Clermont-Ferrand, CHU Clermont-Ferrand, Preventive and Occupational Medicine, WittyFit, Clermont-Ferrand, France; University of Haifa, ISRAEL

## Abstract

**Introduction:**

The COVID-19 pandemic has initiated an upheaval in society and has been the cause of considerable stress during this period. Healthcare professionals have been on the front line during this health crisis, particularly paramedical staff. The aim of this study was to assess the high level of stress of healthcare workers during the first wave of the pandemic.

**Materials and methods:**

The COVISTRESS international study is a questionnaire disseminated online collecting demographic and stress-related data over the globe, during the pandemic. Stress levels were evaluated using non-calibrated visual analog scale, from 0 (no stress) to 100 (maximal stress).

**Results:**

Among the 13,537 individuals from 44 countries who completed the survey from January to June 2020, we included 10,051 workers (including 1379 healthcare workers, 631 medical doctors and 748 paramedical staff). The stress levels during the first wave of the pandemic were 57.8 ± 33 in the whole cohort, 65.3 ± 29.1 in medical doctors, and 73.6 ± 27.7 in paramedical staff. Healthcare professionals and especially paramedical staff had the highest levels of stress (p < 0.001 vs non-healthcare workers). Across all occupational categories, women had systematically significantly higher levels of work-related stress than men (p < 0.001). There was a negative correlation between age and stress level (r = -0.098, p < 0.001). Healthcare professionals demonstrated an increased risk of very-high stress levels (>80) compared to other workers (OR = 2.13, 95% CI 1.87–2.41). Paramedical staff risk for very-high levels of stress was higher than doctors’ (1.88, 1.50–2.34). The risk of high levels of stress also increased in women (1.83, 1.61–2.09; p < 0.001 vs. men) and in people aged <50 (1.45, 1.26–1.66; p < 0.001 vs. aged >50).

**Conclusions:**

The first wave of the pandemic was a major stressful event for healthcare workers, especially paramedical staff. Among individuals, women were the most at risk while age was a protective factor.

## Introduction

The COVID-19 pandemic commenced at the end of 2019 and has been exponential since inception [1]. It is the biggest global health crisis ever experienced in the modern world [2]. The health crisis clearly has an impact on the stress levels of individuals, that can lead to forthcoming public health crisis [3]. Many studies have focused on the stress and concern of healthcare professionals [4–7]. However, studies that compared the stress of physicians to paramedical staff are sparse. The characteristics of pandemic-related stress in a context of such magnitude are new and may present some specific features, such as concerns for the future [8, 9]. Some consequences of this pandemic seem to influence people’s stress levels, such as isolation due to lockdowns and the fear of contagion, which can induce chronic stress [10]. The disruption of professional environments secondary to containment measures has been heterogeneous, [11] forcing workers to interrupt their professional activity while some others maintained regular working routines [11]. This was the case for healthcare professionals who had to continue their work despite the risks inherent to the pandemic [12, 13]. Work is already known as a major source of stress for individuals [14]. Nevertheless, the pandemic-related dimension of occupational stress in healthcare professionals, and particularly between medical doctors and paramedical staff, were not reported to our knowledge. Because of their profession, healthcare professionals have had to maintain or even increase their professional workload [13]. At the onset of the crisis, the lack of known treatment forced healthcare professionals to optimize only symptomatic treatments, isolate patients and provide supportive care [15]. Paramedical staff played a pivotal role in this part of patient care [15]. In the absence of a clearly established "cure", "care" predominated [16]. Certain socio-demographic factors such as gender or age can also influence the level of stress at work and thus represent a risk factor. This is the case among nurses, where women are more stressed than men [17]. Age appears to be a protective factor for all workers during the pandemic and older people have developed specific coping strategies that preserve them from high levels of stress [18]. Showing whether the occupation had an influence on the level of work-related stress could make it possible to better identify the populations at risk to implement targeted actions.

Therefore, the main objective of this study was to assess the work-related stress of healthcare professionals during the covid-19 pandemic. Secondary objectives were to compare the stress of medical doctors with that of paramedical staff, and to evaluate the consequences of personal risk factors such as gender, age etc.

## Methods

### Study design

We conducted an international prospective observational study on the general population during the pandemic period of COVID-19 from March to October 2020. We used a computerized anonymous online questionnaire, accessible through the website COVISTRESS.ORG. The questionnaire was translated into ten languages. The questionnaire was hosted by the University Hospital of Clermont-Ferrand, using the REDCap^®^ software. To facilitate its diffusion, the questionnaire was disseminated by any mean (social media, radio, television, internet, mailing lists, etc.). Respondents were informed of the objective of the survey prior to answering the questionnaire. They were also informed that their data would be used anonymously for research purposes. This study was approved by the South-East VI Ethical Committee of France (Clinicaltrials.gov NCT04538586). The ethics committee waived the need for written consent considering that respondents gave their consent by answering to the questionnaire, and that they could also withdraw at any time.

### Participants

**Fig 1:**
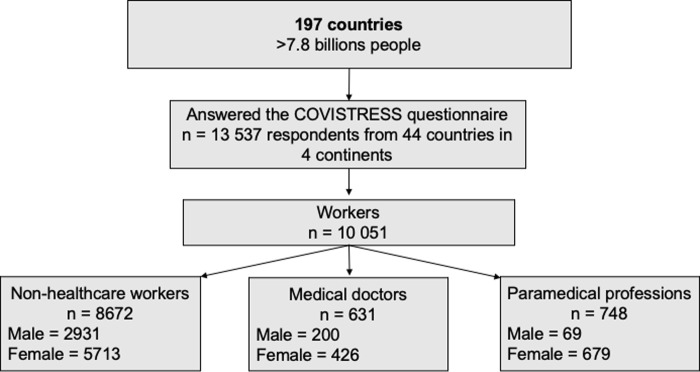
Flow chart. Recruitment characteristics of the study cohort.

The questionnaire was distributed using the international COVISTRESS network. It was disseminated to the general population without distinction of country, gender, occupation or disease (Fig 1).

### Outcomes: Instrument survey

The main outcome was work-related stress, measured with the use of a visual analog scale i.e. a non-calibrated horizontal line ranging from minimum (0) to maximum (100) [19]. Visual analog scale of stress is a validated tool commonly used in daily practice [20, 21]. With this type of tool, participants can self-assess in a simple way the range of their possible feelings [22]. Secondary outcomes were sociodemographic (age, sex), occupations (non-healthcare workers, medical doctors, paramedical staff), and working conditions (working in usual conditions, working in unusual conditions, interruption of work). This was a computerized questionnaire hosted on the secure REDCAP® platform. It consisted of about 100 questions. The study presented here reports on the answers related to work-related stress. Depending on the answers given, individuals had access to all or part of the questionnaire. The questions used for this study are available in S2 Appendix.

### Statistical analysis

**Fig 2:**
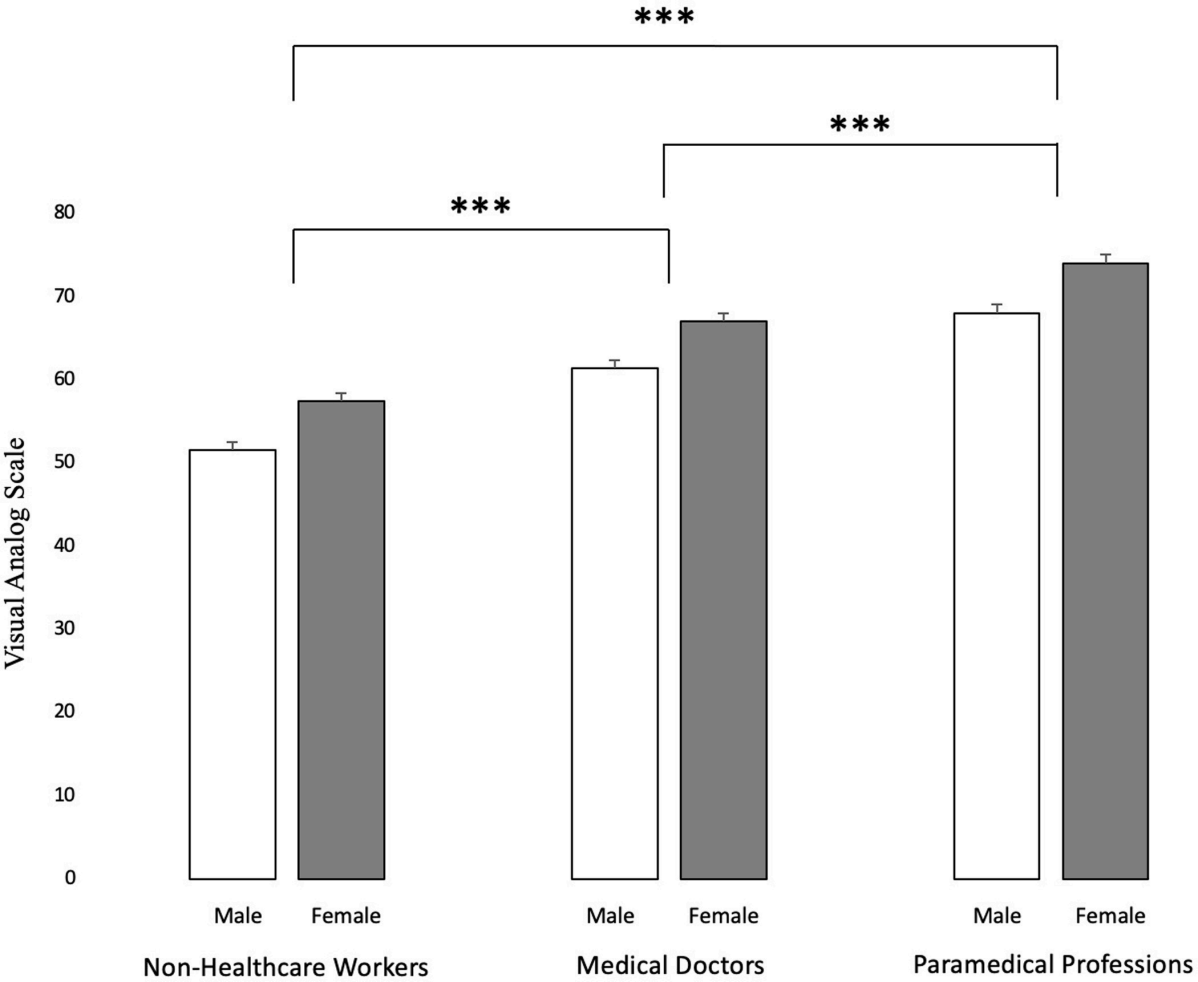
Score of stress at work by occupation and gender. White bars represent male respondents and grey bars female respondents. *** represents p < 0.001.

**Fig 3:**
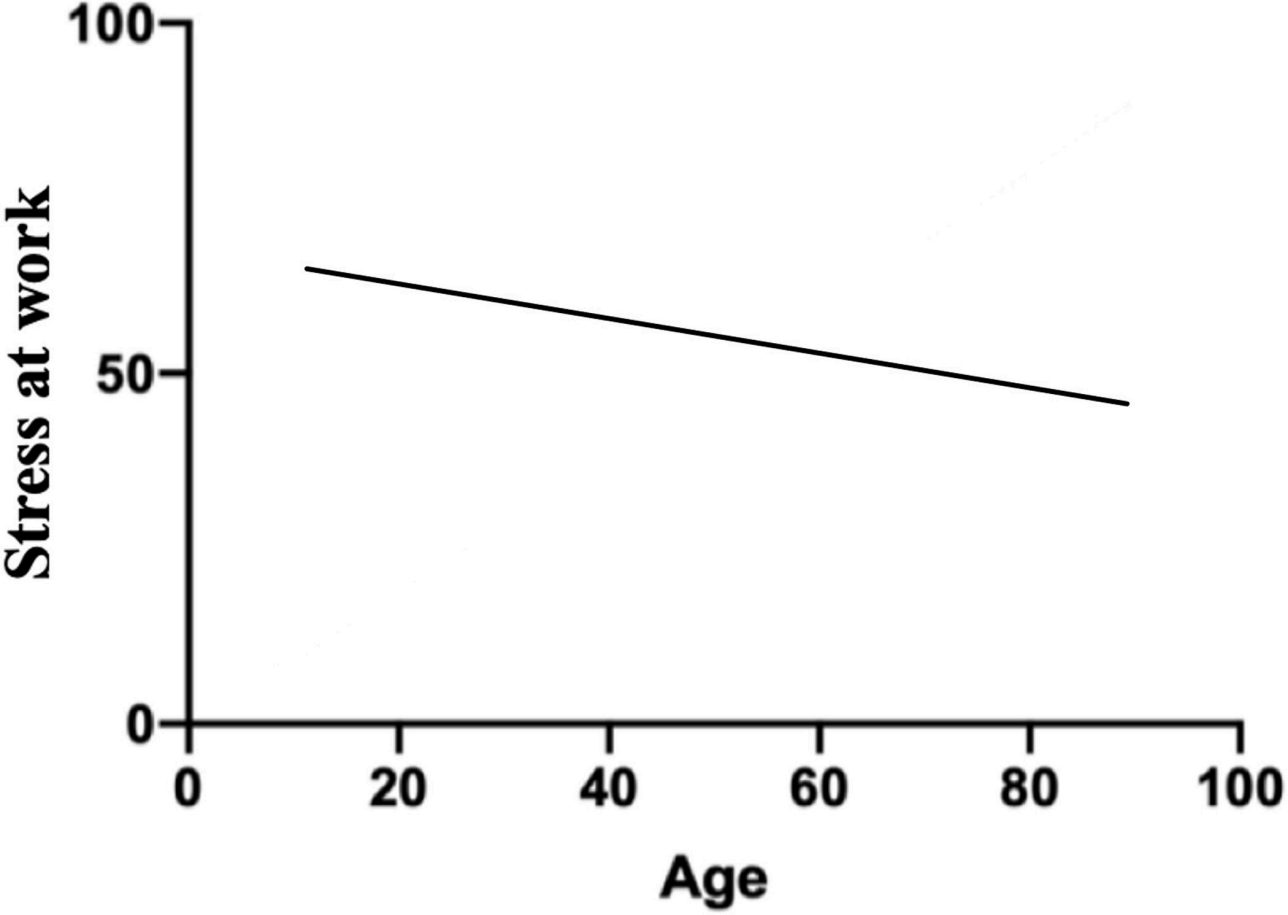
Correlation of age and stress at work.

Data were expressed in number and percentage for categorical variables and mean ± standard deviation (SD) for quantitative variables. Statistics were computed using Stata software (v16, StataCorp, College station, USA). Comparisons between categorical variables were accomplished using Chi2 (χ^2^) and contingency tables. Comparisons between quantitative variables, such as the levels of stress at work according to professional practice during the pandemic, were executed using ANOVA (Fig 2). A Pearson’s r test was carried out to study the correlation between numerical values such as stress levels and age. Logistic regressions were performed to evaluate the risk factors of stress in the workplace (Fig 3). Results were expressed as odds ratio (OR) and 95% confidence intervals (95% CI). Close attention was paid to examining multicollinearity and interactions between covariates: 1) studying the relationships between the covariables, 2) estimating the variance inflation factor and 3) measuring the impact of adding or removing variables in the multivariable model [23, 24]. We first performed univariate regressions for each explanatory factor and then performed a stepwise approach on the status of healthcare worker and on the fact of being a doctor or paramedical staff by adding one by one the other potential explanatory factors (work or not, age and gender).

**Fig 4:**
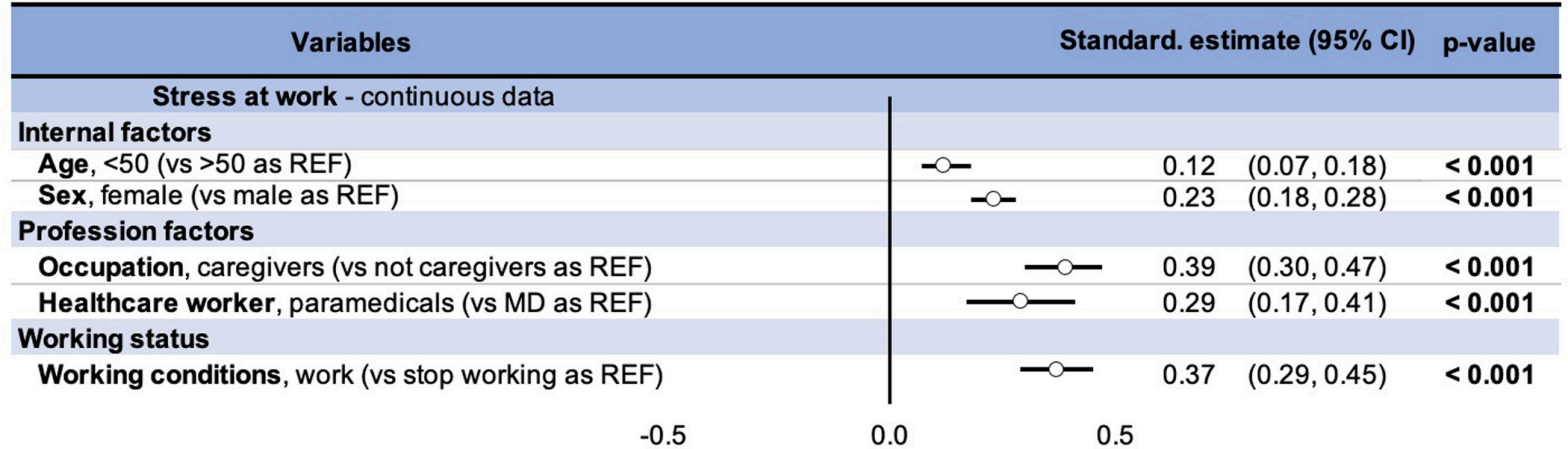
Multivariate analysis of work-related stress. Abbreviations: REF: reference variable, MD: Medical doctors, CI: confidence intervals.

**Fig 5:**
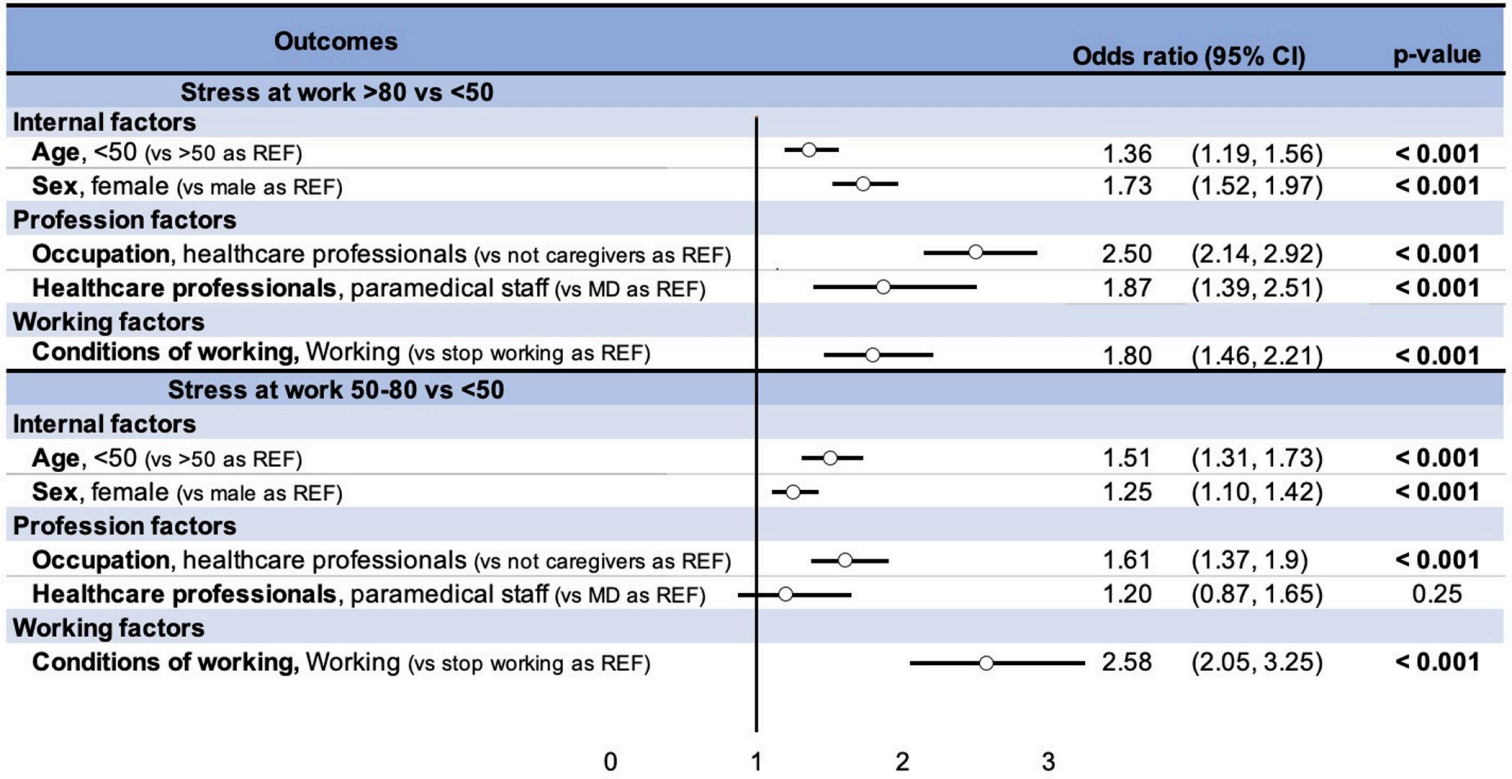
Risk factors for having a score of work-related stress within the ranges 50–80 or >80.

Regression’s analysis were performed to evaluate the risk factors of stress at the workplace. When stress was expressed as a quantitative variable, results were expressed as coefficient and its 95% confidence intervals (95CI) (Fig 4). Close attention was paid to examining multicollinearity and interactions between covariates: 1) studying the relationships between the covariables, 2) estimating the variance inflation factor and 3) measuring the impact of adding or removing variables in the multivariable model. We first performed univariate regressions for each explanatory factor and then performed a stepwise approach on the status of healthcare worker and on the fact of being a doctor or paramedical staff by adding one by one the other potential explanatory factors (work or not, age and gender) (S1 Table). Finally, we ran logistic regressions to quantify the influence of risk factors on « at-risk »stress levels (i.e. in range 50–79 out of 100) and on « intervention » stress levels (i.e. in range 80–100 out of 100). When stress was expressed as a qualitative variable (« at-risk » and « intervention » thresholds), results were expressed as odds ratio (OR) and 95CI (Fig 5). Sensitivity analyses were also conducted for OR (S2 Table). A value of p ≤ 0.05 was needed for statistical significance.

## Results

### Participants

We received 13,547 responses to the questionnaire from 44 countries. The distribution of responses by continent was as follows: Europe 82.8% (n = 9757), America 8.5% (n = 1002), Africa 5,2% (n = 617), Asia 3.4% (n = 404). Due to missing data precluding statistical analysis, 3496 participants were excluded, resulting in a final sample of 10,051 (Fig 1). Respondents were 41.0 ± 14.0 years old; 31.8% were men. There were 8,644 non-healthcare workers, and 1,379 healthcare professionals (631 medicals doctors and 748 paramedical staff). Women were relatively more frequent in healthcare professions than in the general population (χ^2^ = 112, n = 10018; p < 0.001). While lockdown and staying at home was recommended, 90.3% (n = 6,639) of our cohort maintained occupational activity. 96.6% (n = 1,264) of healthcare professionals maintained their professional activity, more than other workers (χ^2^ = 73.7, n = 7,356; p < 0.001). Despite their equality in maintaining their professional activity, physicians were more prone to declare working in unusual conditions than paramedical staff (working conditions, usual vs unusual, p < 0.001, respectively) (Table 1).

**Table 1:** Characteristics of the individuals.

	Non	Healthcare Workers		Medical	Doctors		Paramedical	Professions	
	n = 8 644	Stress at work (mean ± SD)	p	n = 631	Stress at work (mean ± SD)	p	n = 748	Stress at work (mean ± SD)	p
**All**		55.5 ± 33.2			65.3 ± 29.1			73.6 ± 27.7	
**Gender** (n = 10 018)			**< 0.001**			**0.027**			0.09
Female	5 713	57.5 ± 33.2		426	67.1 ± 28.8		679	74.1 ± 27.7	
Male	2 931	51.6 ± 32.8		200	61.4 ± 29.8		69	68.1 ± 28	
**Age** (n = 9 968)			**< 0.001**			**0.001**			0.48
< 30 years	1 932	56.9 ± 33.9		124	62.1 ± 31.7		160	73.3 ± 27.4	
30–50 years	3 721	58 ± 32.3		376	68.9 ± 27.7		450	74.4 ± 27.6	
> 50 years	1 894	48.8 ± 33.4		126	57.4 ± 29.1		135	70.9 ± 28.6	
**Working conditions** (n = 7356)			**< 0.001**			**0.003**			**0.001**
Usual conditions	1 765	60.4 ± 31.7		273	66.7 ± 27.7		392	75.8 ± 26.7	
Unusual conditions	3 205	56 ± 31.9		292	65.5 ± 29.5		264	73.4 ± 26.5	
Stop working	553	46.7 ± 37		27	42.1 ± 39.2		21	53 ± 42.8	

### Main outcome: Work-related stress

The level of work-related stress during the pandemic was influenced by profession (Fig 2). Healthcare professionals had levels of stress 25.8% higher than the general population (p < 0.001). Among healthcare workers, paramedical staff had levels of stress 12.7% higher than medical doctors (p < 0.001). Regardless of occupational category, women were systematically more stressed than men (between 12.2% and 20.7% depending on occupational category, p < 0.001) (Fig 2). Working under unusual conditions did not have an impact on healthcare professionals’ job stress.

### Correlation

Workers’ age was inversely correlated with stress scores (Pearson r = -0.098; p < 0.001), as presented in (Fig 3).

### Univariate and multivariate analyses

Using work-related stress as a continuous variable, being a health professional or paramedical staff, continuing to work, gender, and age were linked with stress scores (p < 0.001). Univariate analyses demonstrated higher scores of work-related stresses in healthcare professionals (Standardized estimate 0.43, 95% CI 0.37 to 0.49). Among healthcare professionals, paramedical staff presented higher stress scores (0.29, 0.18 to 0.39 vs medical doctors). Continuing professional activity (vs. interrupted work), female gender and age < 50 were significantly associated with higher stress scores both in univariate and multivariate analysis, as expressed in (Fig 4). Sensitivity analyses demonstrated that the status of healthcare worker and the fact of being a doctor or paramedical staff was a risk factor of stress whatever the covariates (S1 Table).

### Quantification of risk

Using work-related stress as a qualitative variable, multivariate analyses showed that the risk of very-high level of stress (intervention threshold, >80) was twice the magnitude in healthcare professionals compared to other workers (OR = 2.13, 95% CI 1.87–2.41). Within healthcare professionals, the risk of very-high level of stress (>80) increased by 88% for paramedical staff compared to medical doctors (OR = 1.88, 95%CI 1.50–2.34). The risk of very-high levels of stress (>80) increased in those who continued to work (OR = 1.22, 95%CI 1.01–1.49) compared to those who stopped working), by 83% in women (OR = 1.83, 95%CI 1.61–2.09) compared to men and by 45% in people under 50 years of age (OR = 1.45, 95%CI 1.26–1.66) compared to those who are older). Quantification of risk for those factors was similar for levels of stress above the at-risk threshold (>50) (Fig 5). Sensitivity analyses demonstrated similar findings (S2 Table).

## Discussion

The main findings were that healthcare professionals were the most at risk of stress during the pandemic, globally. Among this population, paramedical staff were more at-risk than physicians. Age and gender also appear to mitigate the experience of work-related stress. Our results are comparable to the literature on the subject [25–27]. Thus, we found higher levels of stress among healthcare professionals compared to other workers. Among healthcare workers, nurses are more stressed than doctors. Women were more affected than men. Young people are a population more at risk of high stress.

### A major stress of healthcare professionals during the first wave

Given the sanitary nature of the COVID-19 global crisis, healthcare professionals have been on the front line dealing with the pandemic [28–30]. Despite their professionalism, overburdened, overworked and under-equipped [31] healthcare systems may account for higher stress among these individuals [32, 33]. It is already known that the pandemic may have led to an increase in burnout among these workers [34, 35]. The chronic stress observed is itself a risk factor for mental [36] and physical [37–39] health issues. The duration and intensity of the pandemic, which was a major source of stress, was the cause of depression among healthcare professionals [40]. Longer lasting and more profound consequences such as post-traumatic stress, suicides, depression have been observed, but as the pandemic is still ongoing, these consequences may be even more important [41, 42]. We were interested here in work-related stress and thus in the short term. However, the consequences of this stress for healthcare workers will persist in the medium and long term [43].

### An even greater stress for paramedical staff

Even if patient care is necessarily multidisciplinary, being a paramedic is an additional risk factor for stress [44]. Paramedical staff appear to have been more exposed than physicians. In the absence of specific COVID-19 treatment, healthcare professionals must provide basic, comfort and symptomatic care. This type of care preferentially involves paramedical staff in contact with the patient and therefore potential contamination [45]. Paramedical staff were more exposed to the lack of material and human resources than physicians [5]. Nurse-to-patient ratio standards in critical care services required new resources during the pandemic. This meant that some paramedical staff were reallocated to understaffed units to provide help and increase manpower [46]. This was also not a trivial issue in terms of work-related stress and was much less the case for physicians who, because of their specialties, remained within their areas of expertise. The sources of stress were thus more important for paramedical staff, including nurses [5]. The high levels of work-related stress among nurses during the pandemic are leading to increased burnout among these health professionals [47]. The effects of the pandemic on covid are major as they increase psychological distress and the desire of nurses to leave the profession [48]. This is an important point because the period requires maximum nursing resources and their departure generates recruitment problems which may lead to the closure of certain beds or even units as may be the case with critical care beds. Another problem resulting from the stress and burnout of nurses is the decline in the quality of care when nurses are affected [49]. This phenomenon could increase in the weeks and months to come, according to the results of the survey carried out by the National Order of Nurses of France [50].

### Women workers might be more affected

In our study, whatever their profession, women had the highest levels of work-related stress during the first global lockdown. Our results concord with the literature revealing that women are more prone to stress [51–53], and may also suffer more from the negative psychological impact of the COVID-19 outbreak [39, 54]. Women often have a double life combining work and family life [55, 56]. This is even less reconcilable when both professional and family constraints increase. Indeed, families had to adapt to the closure of schools. Even in couples that shared the involvement in the education and care of children, women are still mostly implicated [55]. Given these elements and the predominance of women in healthcare professionals, the WHO advised to study gender-specific consequences of the pandemic [57]. Even if women have less severe forms of COVID [58], they were frightened of contracting COVID-19 [54]. They may also have been more impacted by the higher number of deaths and difficulties during the crisis. Women show greater psychophysiological concordance and consistency than men [59], and may therefore present more psychological vulnerability [60, 61].

### Youth are also at risk

The average age of our cohort corresponds to the average age of the population of developed countries [62]. Indeed, age seems to have had a significant impact on the level of stress at work. According to the literature [63], older staff seemed less stressed, despite facing a higher risk of mortality and morbidity from COVID-19 [64]. Our results on a much larger population confirm these findings. A possible explanation may be that younger healthcare professionals are less experienced, which may contribute to an increased work-related stress, especially in complex and/or difficult work situations, such as during the pandemic [65]. The lack of human resources was such that people with little (young graduates …) or no experience (students …) were solicited to come and work in sectors where fully qualified professionals were absent [66]. It was demonstrated that healthy students who were involved during the pandemic had high levels of job stress and indications of mental distress [66]. Individuals in our cohort under 50 years of age were predominantly female, which may explain their high level of work stress, which is more prevalent among younger individuals. The greater feminization of paramedical staff compared to physicians [67] may partly explain this difference, but is not exclusive.

### Limitations

Our study has limitations. We have collected data through a cross-sectional study, a methodology which has its own limitations but allows for a large number of responses [68]. Collecting only declarative data, each individual was able to answer anonymously without any possible control. As a result, the study may be subject to self-reporting bias, especially when questions were omitted by the participants, as well as non-disclosure and uncertainty regarding timing of the questionnaire. Nevertheless, the anonymous nature of the survey may limit biases in the answers. To limit this bias, we have eliminated all incomplete or questionable answers.

Second, some countries and continents are more represented than others, which may limit the generalizability of the data collected. However, this project may be one of the largest studies on this topic due to the number of responses (13,537), and the variety of covered geographic areas, making it more relevant than monocentric studies [69–71]. There could also be measurement bias occurring from the scales used, but this method is scientifically sound [19]. The number of respondents in our study is consistent with the recommendations for this type of analysis [72]. Self-assessed stress levels can be complex because the question may seem vague. However, in a transactional approach, this personal and individual evaluation remains relevant.

This study also presents strengths. Our cohort was representative in terms of age and gender for health professionals even if we did not exhaustively profile these workers (job tenure…) [73]. The international nature of this study may increase the generalizability of our results even if the inhomogeneous distribution of response across the globe generates limits to this external validity. Besides, this study compares stress across professions, which is often a missing element of research regarding stress in healthcare workers. Due to the cross-sectional design of this study, the causal relationship between the risk of work-related stress and mental health needs to be investigated through longitudinal studies.

## Conclusions

The COVID-19 pandemic has and will have consequences for every population. Nevertheless, healthcare professionals were more impacted than other workers by work-related stress. Paramedical staff were more impacted on than physicians. Across all occupational categories, age appears to mitigate work-related stress, and maybe due to the effects of experience. We were able to identify risk factors for high levels of work-related stress such as youth, female gender, paramedical professions and having maintained one’s professional activity. The impact of such a surge in work-related stress may inflict a second blow to already fragile healthcare systems. Adequately monitoring work-related stress and its effects on healthcare workers may be crucial to plan for post-pandemic adjustments.

## Supporting information

S1 Appendix Crude data used and analyzed in the study. (XLSX)

S2 Appendix English version of the questionnaire (questions used for this study). (DOCX)

S1 Table Sensitivity analyses of factors increasing work-related stress. (DOCX)

S2 Table Sensitivity analyses of risk factors for high score of work-related stress: Stepwise approach to the status of healthcare worker. (DOCX)
