# Supplementary material for: The major worldwide stress of healthcare professionals during the first wave of the COVID-19 pandemic – the international COVISTRESS survey
Source: PLoS One. 2021 Oct 6;16(10):e0257840. doi: 10.1371/journal.pone.0257840 (PMC8494302; doi:10.1371/journal.pone.0257840)
Supplement: S2 Appendix — (DOCX) [file pone.0257840.s002.docx]

**COVISTRESS.org**

Through this questionnaire, we would like to study the impact of the **Co**rona**vi**rus on your life and on your **stress**, particularly on work and personal life. Even if you have not had contact with a person carrying the virus, we are interested in the impact that this epidemic may have on your life. This study is an international collaboration between several Institutions (University Hospitals, Universities, CNRS, and Occupational Health).

Thank you for your participation*.* [Access the questionnaire](https://redcap.chu-clermontferrand.fr/surveys/?s=MNL4WW7WH8) [Download pdf](http://covistress.org/pdfs/2020-03-30_Flyer_COVISTRESS.pdf)

To access the survey, type into the search field: [**COVISTRESS.org**](http://covistress.org/)

Or <https://redcap.chu-clermontferrand.fr/surveys/?s=MNL4WW7WH8>


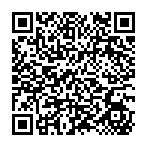
Or scan the following QR code:

**Questionnaire used for this study**

**In which country do you work?**

**Gender** □ Female □ Male

**Age:**

**What is your level of…?**

… stress at home

Minimum

Maximum

… stress at work

Minimum

Maximum

**Actual status: Health-care workers:** □ **yes** □ **no**

**🡪 If yes:** □**Medical** **doctors** □**Paramedical staff**

**During the lockdown, what has happened to your professional activity?**

□ **Stop working** (work stoppage or specific leave of absence etc.) □ **Keep working**

**🡪 If keep working, what were your working conditions:**  Usual conditions □ **yes** □ **no**
