# Supplementary material for: The major worldwide stress of healthcare professionals during the first wave of the COVID-19 pandemic – the international COVISTRESS survey
Source: PLoS One. 2021 Oct 6;16(10):e0257840. doi: 10.1371/journal.pone.0257840 (PMC8494302; doi:10.1371/journal.pone.0257840)
Supplement: S1 Table — (DOCX) [file pone.0257840.s003.docx]

|  | **1 factor** | **2 factors** | **3 factors** | **4 factors** |
| --- | --- | --- | --- | --- |
| **Age, <50**  (vs >50 as REF) | 0.28 (0.23 to 0.32)  p<0.001 | 0.26 (0.21 to 0.30)  p<0.001 | 0.24 (0.20 to 0.29)  p<0.001 | 0.14 (0.09 to 0.20)  p<0.001 |
| **Sex**, **female**  (vs male as REF) |  | 0.21 (0.16 to 0.25)  p<0.001 | 0.17 (0.12 to 0.21)  p<0.001 | 0.21 (0.15 to 0.26)  p<0.001 |
| **Occupation, healthcare worker** (vs not healthcare worker as REF) | - |  | 0.39 (0.33 to 0.45)  p<0.001 | 0.35 (0.29 to 0.41)  p<0.001 |
| **Working conditions, work**  (vs stop working as REF) |  |  |  | 0.39 (0.30 to 0.47)  p<0.001 |

**Sensitivity analyses of factors increasing work-related stress**
