# Supplementary material for: The major worldwide stress of healthcare professionals during the first wave of the COVID-19 pandemic – the international COVISTRESS survey
Source: PLoS One. 2021 Oct 6;16(10):e0257840. doi: 10.1371/journal.pone.0257840 (PMC8494302; doi:10.1371/journal.pone.0257840)
Supplement: S2 Table — (DOCX) [file pone.0257840.s004.docx]

|  | **1 factor** | **2 factors** | **3 factors** | **4 factors** |
| --- | --- | --- | --- | --- |
| **Occupation, healthcare worker** (vs not healthcare worker as REF) | -13.6 (0.07 to 0.18); p<0.001 | 0.12 (0.07 to 0.18); p<0.001 | 0.12 (0.07 to 0.18); p<0.001 | -11.3 (1.00 to 0.18); p<0.001 |
| **Working conditions, work**  (vs stop working as REF) |  | 0.12 (0.07 to 0.18); p<0.001 | 0.12 (0.07 to 0.18); p<0.001 | -12.54 (0.07 to 0.18); p<0.001 |
| **Age, <50**  (vs >50 as REF) |  |  | 0.12 (0.07 to 0.18); p<0.001 | -4.59 (0.07 to 0.18); p<0.001 |
| **Sex**, **female**  (vs male as REF) |  |  |  | -6.66 (0.07 to 0.18); p<0.001 |

**Sensitivity analyses (stepwise approach) of risk factors for having a score of work-related stress within the ranges 50-80 or >80**

**Sensitivity analyses (stepwise approach) of risk factors for being a healthcare worker medical doctor or paramedical staff**

|  | **1 factor** | **2 factors** | **3 factors** | **4 factors** |
| --- | --- | --- | --- | --- |
| **Healthcare worker, Paramedical**  (vs Medical as REF) | -8.83 (0.07 to 0.18); p<0.001 | -8.87 (0.07 to 0.18); p<0.001 | -8.72 (0.07 to 0.18); p<0.001 | -7.31 (0.07 to 0.18); p<0.001 |
| **Working conditions, work**  (vs stop working as REF) |  | -22.8 (0.07 to 0.18); p<0.001 | -22.9 (0.07 to 0.18); p<0.001 | -23.3 (0.07 to 0.18); p<0.001 |
| **Age, <50**  (vs >50 as REF) |  |  | -5.57 (0.07 to 0.18); p=0.008 | -5.68 (0.07 to 0.18); p=0.006 |
| **Sex, female**  (vs male as REF) |  |  |  | -6.15 (0.07 to 0.18); p=0.003 |
